# Redescription of *Mymarilla* Westwood, new synonymies under *Cremnomymar* Ogloblin (Hymenoptera, Mymaridae) and discussion of unusual wings

**DOI:** 10.3897/zookeys.345.6209

**Published:** 2013-10-29

**Authors:** John T. Huber

**Affiliations:** 1 Natural Resources Canada, c/o Canadian National Collection of Insects, AAFC, K.W. Neatby building, 960 Carling Avenue, Ottawa, ON, K1A 0C6, Canada

**Keywords:** Mymaridae, *Mymarilla wollastoni*, *Cremnomymar*, *Richteria*, *Parapolynema*, wing modifications, island faunas

## Abstract

The monotypic genus *Mymarilla* Westwood is known only from St. Helena, a remote island in the South Atlantic Ocean. The peculiar species *M. wollastoni* Westwood (Mymaridae) is redescribed and illustrated from non-type material. *Mymarilla* is compared with *Cremnomymar* Ogloblinspp. from the Juan Fernández Islands in the South Pacific Ocean. *Stephanodes* Enock is shown to be the most likely sister genus to *Mymarilla*. *Nesopolynema* Ogloblin, **syn. n.**, *Oncomymar* Ogloblin, **syn. n.**, *Scolopsopteron* Ogloblin, **syn. n.**, are placed in synonymy under *Cremnomymar* and their species transferred as *Cremnomymar caudatum* (Ogloblin 1952), **comb. n.**, *C. dipteron* (Ogloblin 1957), **comb. n.**, and *C. kuscheli* (Ogloblin 1952), **comb. n.** Wing shape and wing reductions in Mymaridae are discussed in relation to biogeography, particularly with respect island faunas and to four genera, *Cremnomymar*, *Mymarilla*, *Parapolynema* Fidalgo, and *Richteria* Girault, some or all of whose species have more or less convex fore wings.

## Introduction

The small, remote South Atlantic island of St. Helena contains a high proportion of endemic species but among the Hymenoptera only the Formicidae have been systematically studied (Wetterer et al. 2007). Among the insects, one of the most remarkable is *Mymarilla wollastoni* Westwood (Hymenoptera: Mymaridae). Although the species was described briefly from both sexes (Westwood 1879), it is instantly recognizable from the original illustrations that show the peculiar, strongly convex fore wings. Annecke and Doutt (1961) redescribed the species in greater detail, designated a lectotype, and cleared up the confusion by previous authors over what species should be included in *Mymarilla* Westwood. Subba Rao (1976) illustrated the species with a habitus drawing. *Mymarilla* is redescribed from material other than the original type series and is compared with new material of *Cremnomymar* Ogloblin and its synonyms (proposed below) from the Juan Fernández Islands, about 600 km W. of Chile in the South Pacific Ocean. Wing shape, and wing reduction and/or convexity is discussed in relation to geographical distribution of Mymaridae.

## Methods

Morphological terms follow Gibson (1997) and Huber (2012). Measurements are in micrometres (µm). Photographs of critical point dried and card mounted specimens were taken with a ProgRes C14^plus^ digital camera attached to a Nikon Eclipse E800 compound microscope, and the resulting layers combined electronically using Auto-Montage® or Zyrene Stacker® and retouched as needed with Adobe® Photoshop CS6®. Specimens of *Cremnomymar* were gold coated for scanning electron micrography, using the techniques described in Bolte (1996).

Specimens are in the following institutions.

BMNH The Natural History Museum (formerly British Museum [Natural History]), London, England (G. Broad).

CNC Canadian National Collection of Insects, Ottawa, Canada.

MRAC Musée Royal de l’Afrique Centrale, Tervuren, Belgium (E. de Coninck).

OUMNH Oxford University Museum of Natural History.

## Systematics

### Mymarilla

Westwood

http://species-id.net/wiki/Mymarilla

Mymar : Westwood 1879: 585 (*Mymarilla wollastoni* included, together with another, correctly placed species).
Mymarilla Westwood, 1879: 585 (footnote) + figs 8, 9 (recommended as a new genus group name for *Mymarilla wollastoni* “if it should be deemed necessary to separate this species from the genus *Mymar*”).
Mymar : Dalla Torre 1898: 427 (*Mymarilla* treated as a synonym).
Mymarilla : Schmiedeknecht 1909: 495 (treated as valid genus with one [incorrectly placed] species but no mention of *Mymarilla wollastoni*).
Mymar : Schmiedeknecht 1909: 496 (listed *M. Wollastoni* [*sic*] together with three other species [one other of which is also incorrectly placed generically]).
Mymarilla : Ferrière 1952: 43 (treated as valid name for *Mymar* of authors, not Curtis).
Mymarilla : Doutt 1955: 11 (key), 12 (treated as valid genus, but noted that American authors used *Mymar* as the name for species included under *Mymarilla* by previous workers).
Mymarilla : Heqvist 1960, 432 (treated as valid genus with one [incorrectly placed] species but no mention of *Mymarilla wollastoni*).
Mymarilla : Annecke and Doutt 1961: 31 (discussion of past confusion with *Mymar*).

#### Type species.

*Mymarilla wollastoni* Westwood, by monotypy. Transferred (as genotype) to *Mymarilla* by Heqvist (1960: 432).

The confusion in the use of the name *Mymarilla* and which species should be placed in the genus continued for over 80 years. Heqvist (1960) was the first to treat *Mymarilla wollastoni* as the genotype of *Mymarilla*, even as he incorrectly placed *Mymar* species under it, as did previous authors. As Annecke and Doutt (1961) clarified, wherever previous authors use *Mymarilla* it was clearly in the sense of *Mymar*, because the species names mentioned are typical *Mymar* species.

#### Diagnosis.

The combination of smooth, shiny black body, extremely short mesocutum compared to much longer pronotum, and extraordinary convex and densely setose fore wing distinguish the genus and species from any other Mymaridae.

*Mymarilla* belongs clearly to the *Polynema* Halidaygroup of genera within Mymarini sensu Annecke and Doutt (1961). They suggested that *Mymarilla* was most similar to *Oncomymar* Ogloblin from the Juan Fernández Islands. Superficially, the most similar genus is *Cremnomymar* Ogloblin (including *Oncomymar* Ogloblinand *Scolopsopteron* Ogloblin, see below), some of whose species also have a convex fore wing and reduced mesoscutum. The two genera are not closely related; their resemblance is due to adaptations to life on remote, presumably wind-swept, oceanic islands.

**Figures 1, 2.**
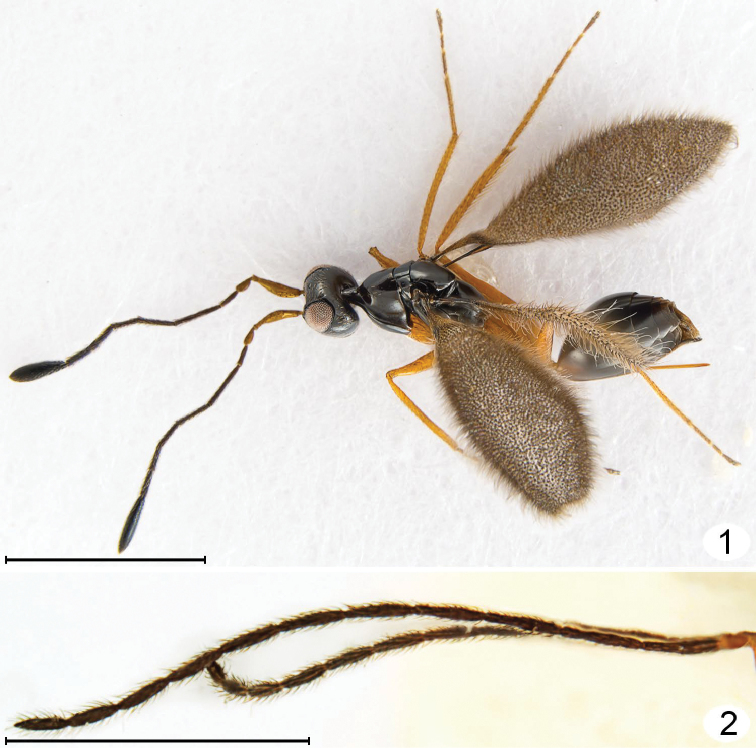
*Mymarilla wollastoni*. **1** female dorsolateral **2** male antennae (pedicel + flagellum). Scale line = 1000 μm.

**Figures 3, 4.**
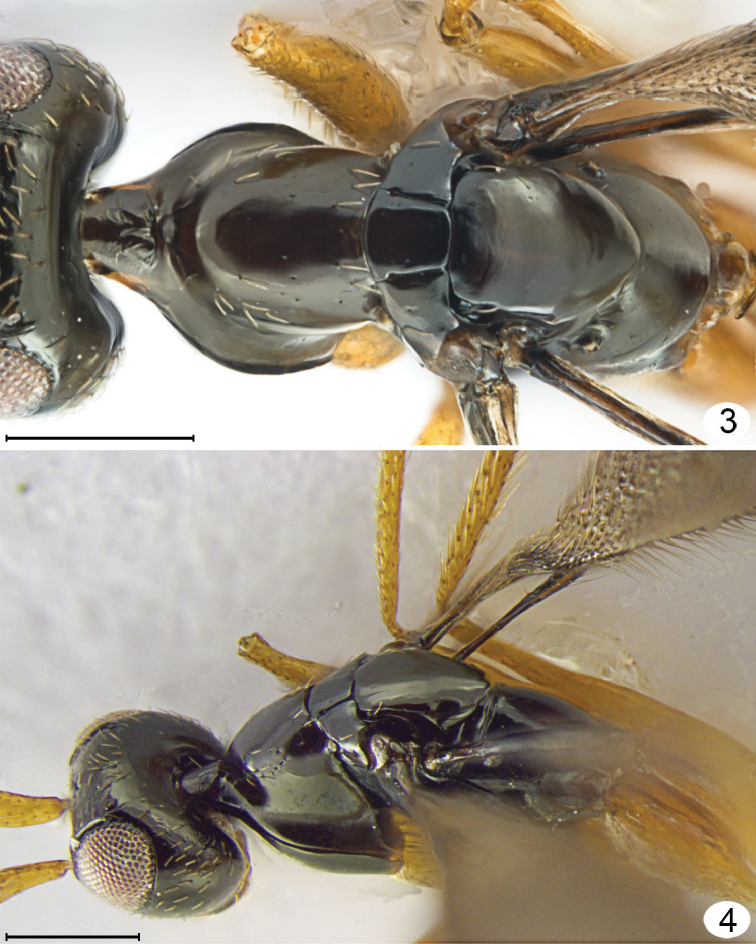
*Mymarilla wollastoni*. **3** mesosoma, dorsal **4** head and mesosoma, dorsolateral. Scale line = 200 μm.

I propose instead that *Mymarilla* is derived from *Stephanodes* Enock, likely the most closely related genus. Four features, shared with *Stephanodes*, suggest this: first, the extremely smooth body without trace of microscupture on the mesosoma (Figs 1, 3, 4, 6, 9, 10); second, the slightly advanced mesothoracic spiracle about midway between the anterior apex of a notaulus and posterolateral angle of the mesoscutum (Figs 3, 4); third, the presence of a metapleural pit (Figs 9, 10); fourth, the fore wings that are held more or less horizontally. In dead specimens of *Stephanodes*, the fore wings are often horizontal, crossed scissor-like and covering the body, unlike other, related genera in the *Polynema*-group where the wings (in dead specimens) are almost always vertical, directed away from the body. The strong convexity of the fore wings of *Mymarilla* would appear to prevent them from being crossed scissor-like over the body. Yet they are presumably capable of enveloping the metasoma, as pointed out by Westwood who noted “... when shut [the fore wings] form a semiglobular dome over the abdomen” when the wasp is at rest. The densely hairy wing membrane with dark base around each microtrichia would allow for maximum heat absorption and retention.

**Figures 5, 6.**
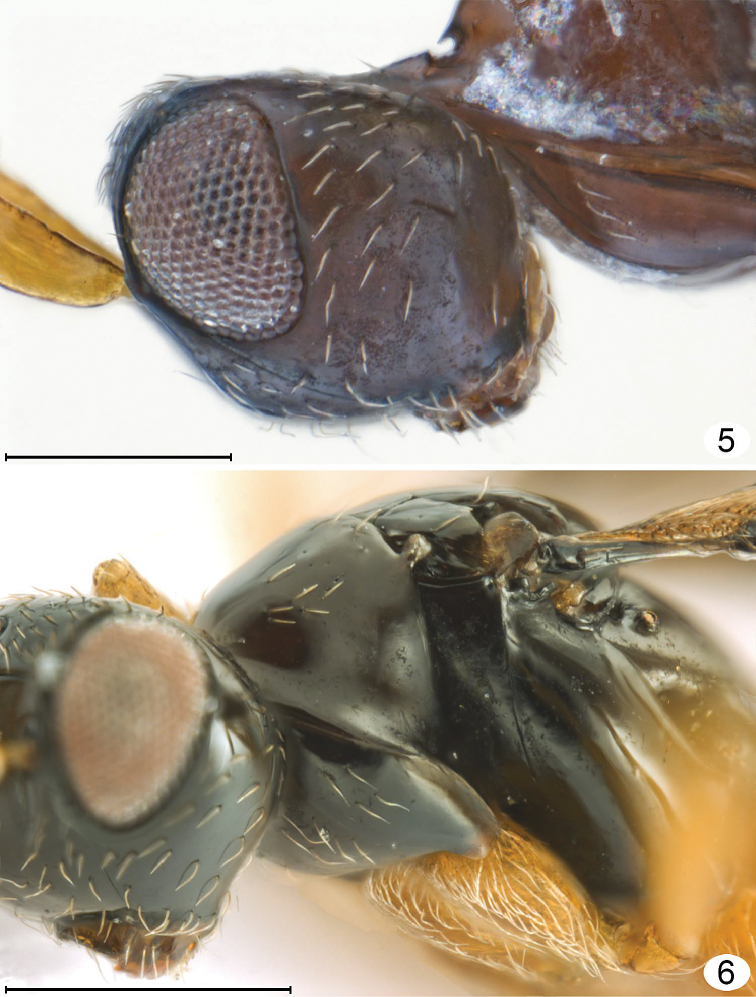
*Mymarilla wollastoni*. **5** head and prothorax, lateral **6** head and thorax, anterolateral. Scale line = 200 μm.

### Mymarilla wollastoni

(Westwood)

http://species-id.net/wiki/Mymarilla_wollastoni

Figs 1 –10

Mymar wollastoni : Westwood 1879: 585 (original desciption).
Mymar wollastonii [*sic*]: Dalla Torre 1898: 427 (catalogue, unjustified emendation).
Mymarilla wollastoni : Annecke and Doutt 1961: 31 (redescription).
Mymarilla wollastoni : Subba Rao 1976: 90 (diagnosis), 91 (figure).

#### Remarks.

Lectotype female in OUMNH (not examined), designated by Annecke and Doutt (1961), with catalogue number HYME0029 and labeled: “Holotype”, “det. D.P. Annecke 25.vii.1960”.

**Figures 7, 8.**
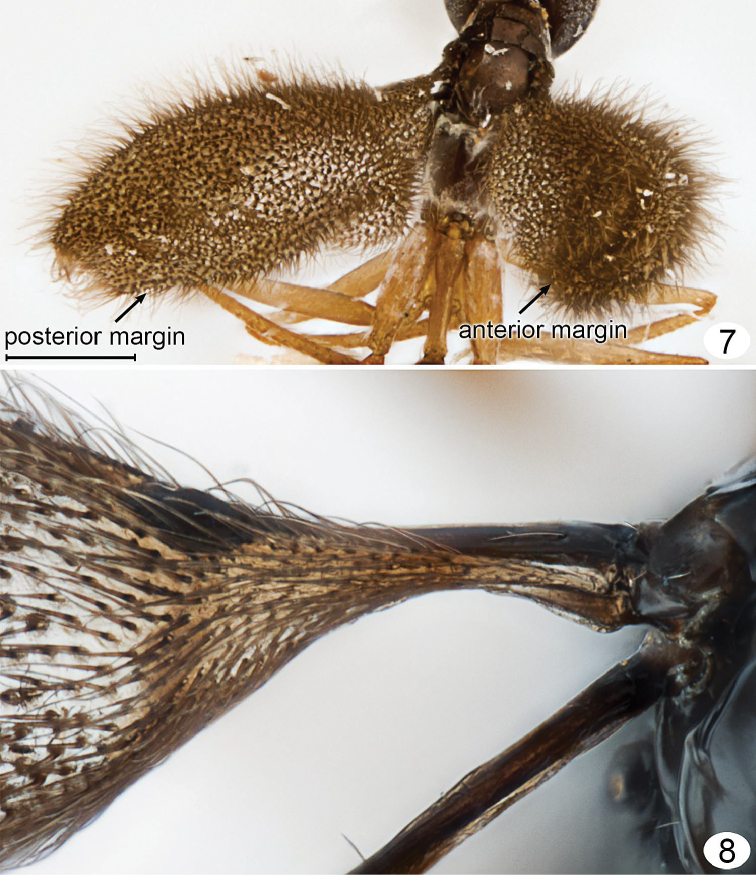
*Mymarilla wollastoni*. **7** mesosoma, petiole and wings posterodorsal **8** fore and hind wing bases, dorsal. Scale line = 200 μm.

**Figures 9, 10.**
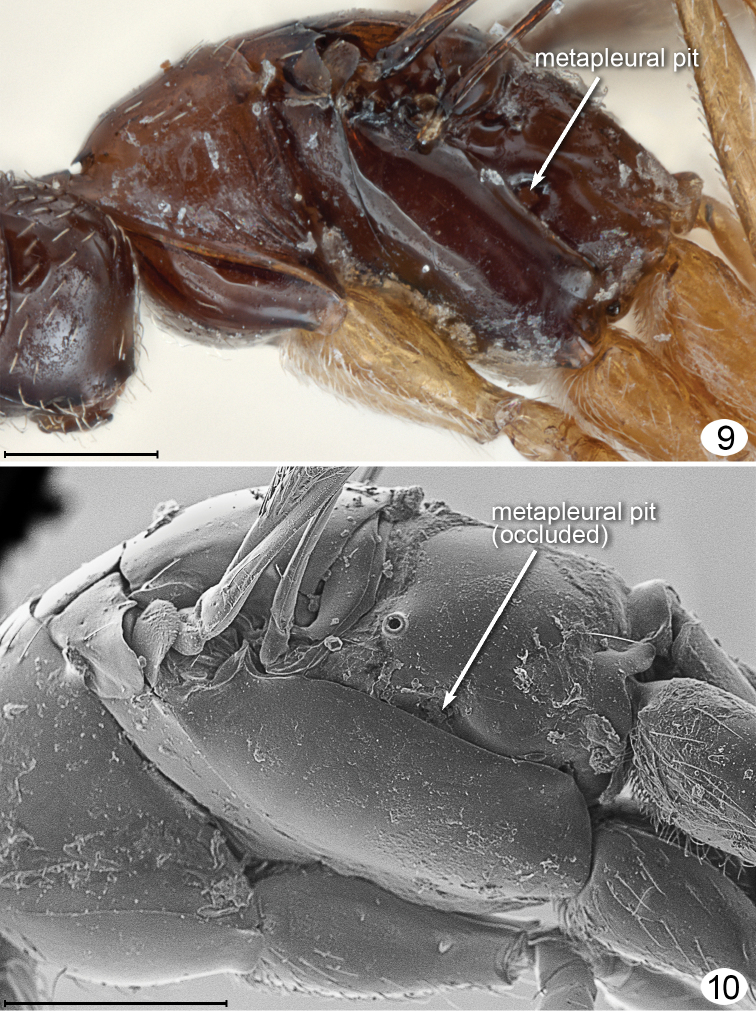
*Mymarilla wollastoni*. **9** mesosoma lateral **10** mesosoma, lateral, SEM. Scale line = 200 μm.

**Description.** Because only one species of *Mymarilla* is known, the generic and specific features are both described in the species description. **Female.** Body length 2125 (n=1). Body entirely smooth and shiny except for a few small punctures on pronotum. **Colour.** Head black except mandibles, mesosoma, metasoma medially, and clava; flagellum, and metasoma basally and apically dark brown; scape, pedicel, mandibles, legs, petiole, and ovipositor brownish yellow; all body setae almost white; wings brown behind venation, membrane beyond venation translucent but membrane around each microtrichia back, so wings generally appearing dark. **Head.** (Figs 3–6) 1.46× as wide as long, and 1.17× as wide as high (406/227/347) (n=1), in lateral view with anterior surface slightly depressed between toruli then, at level of ventral margin of eye, receding to mouth (Fig. 5). Face measured from eye to eye below toruli about as long as wide, flat above toruli but forming a shallow circular depression medially below toruli; subantennal groove and pits between toruli absent. Torulus about 1.5× its diameter from thin transverse trabecula and about mid height of eye. Preorbital groove against eye almost to ventral margin of eye (Fig. 5), then extending almost straight to anterolateral angle of mouth margin. Eye (Fig. 5) in lateral view 0.75× as long as high, not extending to back of head dorsally and separated by more than its own length from back of head ventrally. Malar sulcus absent and malar space almost 0.6× eye height. Gena narrow dorsally, very wide ventrally. Vertex in lateral view almost flat, sloping anteriorly, forming an obtuse angle to face (separated by transverse trabecula), smoothly merging posteriorly with occiput. Mid ocellus flat in shallow depression, not projecting above surface of vertex and 2× diameter of small lateral ocellus. Ocelli in high triangle, with POL (168), 1.9× LOL, and LOL (89) 3.0× OOL (30). Mandibles normal, overlapping medially when closed, with 3 teeth. **Antenna.** Scape about 3.9× as long as wide, with both inner and outer surfaces apparently smooth, and with radicle short but distinct; pedicel slightly shorter than fl_1_, about 0.41× scape length; funicle 6-segmented; clava unsegmented, slightly longer than scape. Number of mps of funicle segments and clava uncertain (not clearly visible on card mounted specimen). Measurements (n=1) length/width: scape 267/69, pedicel 109/50, fl_1_ 129/20, fl_2_ 267/20, fl_3_ 198/30, fl_4_ 149/30, fl_5_ 149/30, fl_6_ 149/30, clava 317/80. **Mesosoma.** About 2.67× as long as wide, about 1.80× as long as high, and 1.48× as wide as high (n=1). Pronotum (Fig. 3) in dorsal view clearly visible, shorter than wide (218: 267), strongly convex, with almost vertical sides flaring outward ventrally and almost horizontal at junction with propleura; pronotum in lateral view strongly triangular. Propleura (Fig. 4) in dorsolateral view tightly pressed to pronotum laterally and anteriorly, and fused to each other at neck, and, in dorsal view (Fig. 3), slightly extending lateral to pronotum. Neck long (90), clearly separating head from pronotum. Prosternum triangular, strongly appressed laterally to propleura, without median longitudinal line, anterior apex not visible but perhaps closed anteriorly. Mesonotal spiracle (Fig. 6) small, at end of short tube, midway between posterolateral angle of mesoscutum and anterior apex of notaulus. Mesoscutum (Fig. 3) smooth and shiny, in lateral view slightly convex, in dorsal view short (99), about 0.4× as long as pronotum and 0.4× as long as wide, with slightly diverging, almost straight notauli, each ending anteriorly in a distinct pit. Axillae not advanced (Figs 3, 4, 9, 10). Mesoscutellum (Fig. 3) almost as long as pronotum (267: 246), slightly overlapping metanotum, without trace of frenal line (frenum therefore not distinguishable). Prepectus (Figs 6, 9, 10) triangular, about 3× as long as dorsal width. Mesopleuron tightly appressed to prepectus, convex, not divided by suture into mesepisternum and mesipimeron. Metanotum small, triangular, separated from propodeum by wide groove. Metapleuron with a large metapleural pit at junction with mesoepisternum. Propodeum (Figs 3, 4, 7, 10) evenly convex, without carinae, with a large pit at anterior margin just anterior to spiracle, with a slightly upturned nucha covering anterior apex of petiole, and with propodeal seta almost at posterior margin. Spiracle small, round, separated by several diameters from anterior margin of propodeum. **Wings.** Fore wing (Figs 1, 7, 8) deeply convex, with the anterior and posterior margins strongly curving downward, the wing *height* in lateral view about 0.7× wing width in dorsal view. Wing behind submarginal vein very narrow, with strongly convex hind margin, abruptly widening beginning at parastigma, generally oval in dorsal view. Entire surface to wing base covered in long microtrichia, those behind venation appressed and those beyond venation semi-erect to erect. Fore wing length 1792 (n=1), width 640, length/width 2.8, venation length 287, about 0.18× forewing length. Submarginal vein black basally, brown apically, and much wider basally than apically; parastigma + stigma black, oval about 1.7× as wide as base of submarginal vein. Costal cell extremely narrow. Hind wing (Fig. 1) flat to slightly convex, with long marginal setae and erect microtrichia similar to those on forewing, and wing membrane extending almost to base of wing but very narrow behind venation. Hind wing length ca. 1535, width 77, venation black, length about 0.3× wing length. **Legs.** Long and slender (Figs 1, 7). **Metasoma.** Petiole length 180, slightly longer than metacoxa, about 6× as long as wide. Gaster (Fig. 1) smooth and shiny, narrowly oval in cross section (as seen in posterior view), wider dorsally, almost knife-like ventrally at ovipositor. Gt_1_ about 0.57× gaster length (870) and almost completely covering gs_1_ so petiole apparently attached to tergum, gt_2_–gt_5_ progressively shorter, gt_6_ and syntergum (gt_7_ + gt_8_) each about as long as gt_2_. Spiracle present on gt_6_, small. Ovipositor length ca. 770, slightly down turned apically, as long as gaster but not exserted beyond gaster, about 1.2× metatibia length (640).

**Male.** Colour as in female but body dark brown (possibly due to fading), scape brownish yellow, pedicel light brown, flagellum dark brown. Body length 1843–1894 (n=2). Fore wing length 1664 (hind wing not measurable on pinned specimens), with the edges almost meeting ventrally in one specimen, giving the appearance of a hirsute cigar when seen end on in posterior view (Fig. 7). **Antenna.** Scape about 3.8× as long as wide, with both inner and outer surfaces apparently smooth. Measurements (n=1) length/width or length for flagellomeres: scape 218/59, pedicel 99/45, fl_1_ 277, fl_2_ 267, fl_3_ 248, fl_4_ 248, fl_5_ 223, fl_6_ 228, fl_7_ 198, fl_8_198, fl_9_ 178, fl_10_ 198, fl_11_ 198; total flagellum 2461. Fl_6_ length/width about 6.0, with perhaps 8 mps (not clearly visible on card mounted specimen). **Metasoma.** Gaster length 742–793 (n=2), in lateral view truncate apically.

#### Material examined.

**SAINT HELENA**. Centre. High Central Ridge, Cabbage Tree Road, 2500’, iii.1967, J. Decelle, N. & J. Leleup (2 males, MRAC); High Peak, 15°58.7'S, 5°44.0'W, ca.752m, xii.2005–1.2006, N.P. & M.J. Ashmole, H. Mendel, E.A. Thorpe, pitfall trap (1 female, BMNH).

#### Habitat.

Westwood (1879) stated that the specimens were swept from low herbage.

Annecke and Doutt (1961) suggested that the wings may be used for floating on air currents. Whether individuals are capable of this, let alone normal flight, is uncertain. The greatly reduced mesoscutum suggests that the flight muscles are so reduced they would be incapable of powered flight. The collection of one female in a pitfall trap suggests that *Mymarilla wollastoni* lives near the ground.

### Cremnomymar

Ogloblin

http://species-id.net/wiki/Cremnomymar

Figs 11 –14 , 20 –38

Cremnomymar Ogloblin, 1952: 120 (generic description, two species described, based on males); Ogloblin 1957: 418 (two species described, based on females); Annecke and Doutt 1961: 6 (key), 31 (comments); Fidalgo, 1982: 98 (comparison with *Parapolynema*);
Scolopsopteron Ogloblin, 1952: 127 (generic description, one species based on two males); Annecke and Doutt, 1961: 6 (key), 30 (comments). **syn. n.**
Nesopolynema Ogloblin, 1952: 132 (generic description, one species based on a male); Annecke and Doutt 1961: 6 (key), 30 (comments). **syn. n**.
Oncomymar Ogloblin, 1952: 132 (generic description, one species based on a female); Annecke and Doutt 1961: 6 (key), 30 (comments). **syn. n.**

#### Remarks.

Ogloblin (1952, 1957) described seven species of Mymaride based on eight specimens from the Juan Fernández Islands, all but one from Masatierra Island (Robinson Crusoe Island). Three were described from females only and four from males only. *Scolopsopteron dipteron* Ogloblin (Ogloblin 1952) has a fore wing of normal length but unusually narrow and a rudimentary hind wing. *Oncomymar kuscheli* Ogloblin (Ogloblin 1957) has a fore wing of normal width and length but strongly convex (spoon-shaped) and a rudimentary hind wing. *Nesopolynema* has a normal fore wing (hind wings missing in Ogloblin’s specimen).

It is improbable that the Juan Fernández Islands would have four endemic genera of Mymaridae, as treated by Ogloblin (1952, 1957). Above the species category similarities must be used to define collective groups, not differences. If differences are used, then how different must something be to be placed in a different genus? “Different enough” is not an acceptable answer. If it were, the placement of species in genera of finer and finer difference would be the norm, with the result that the genus category would become almost synonymous with the species category, and each genus would contain only one or two species. Unfortunately, Ogloblin sometimes defined genera based on obvious but superficial differences that resulted in oversplitting. I am confident that all seven of Ogloblin’s species from the Juan Fernández Islands represent at most different species within a single genus. Indeed some of them have likely been described twice, once from females and once from males.

**Figure 11.**
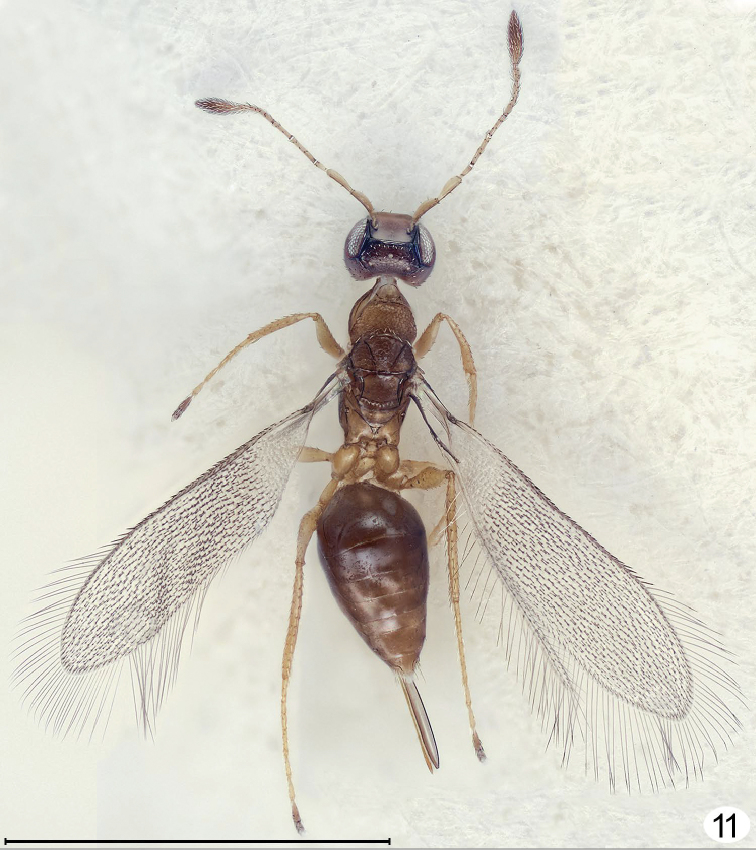
*Cremnomymar* sp., macropterous female, dorsal (fore wing flat). Scale line = 1000 μm.

**Figure 12.**
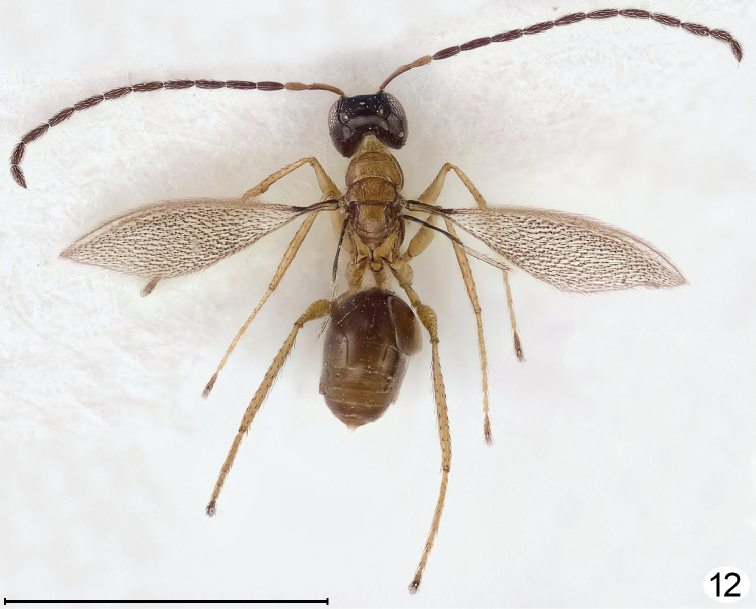
*Cremnomymar* sp., slightly brachypterous male, dorsal (forewing slightly convex, hind wing brachypterous). Scale line = 1000 μm.

**Figure 13.**
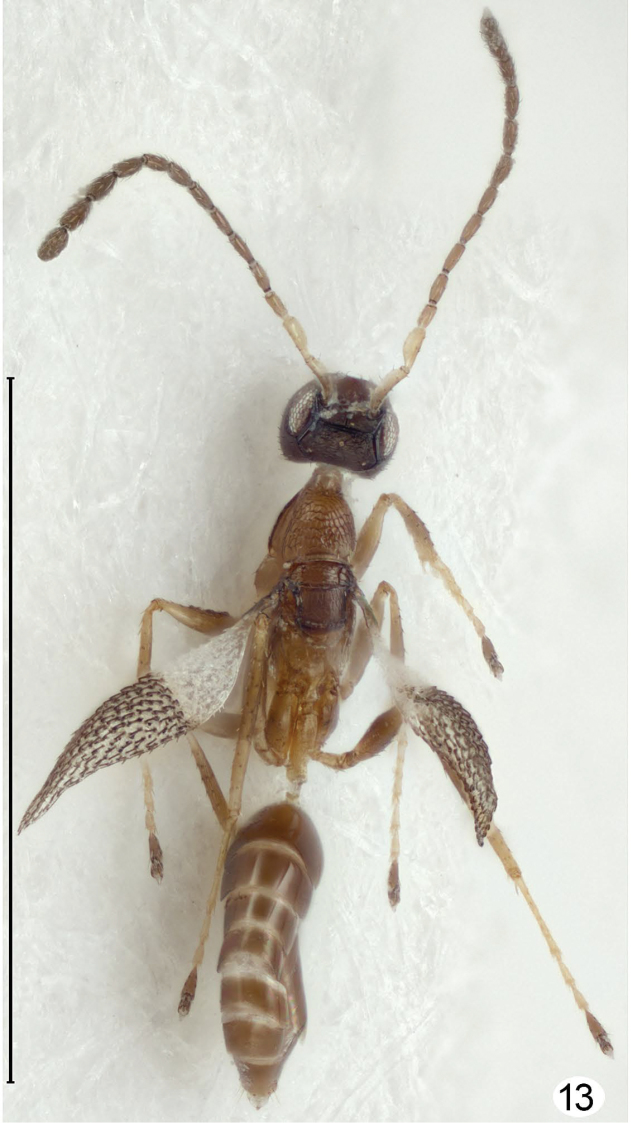
*Cremnomymar* sp., moderately brachypterous male, dorsal (forewing distinctly convex, hind wing micropterous). Scale line = 1000 μm.

**Figure 14.**
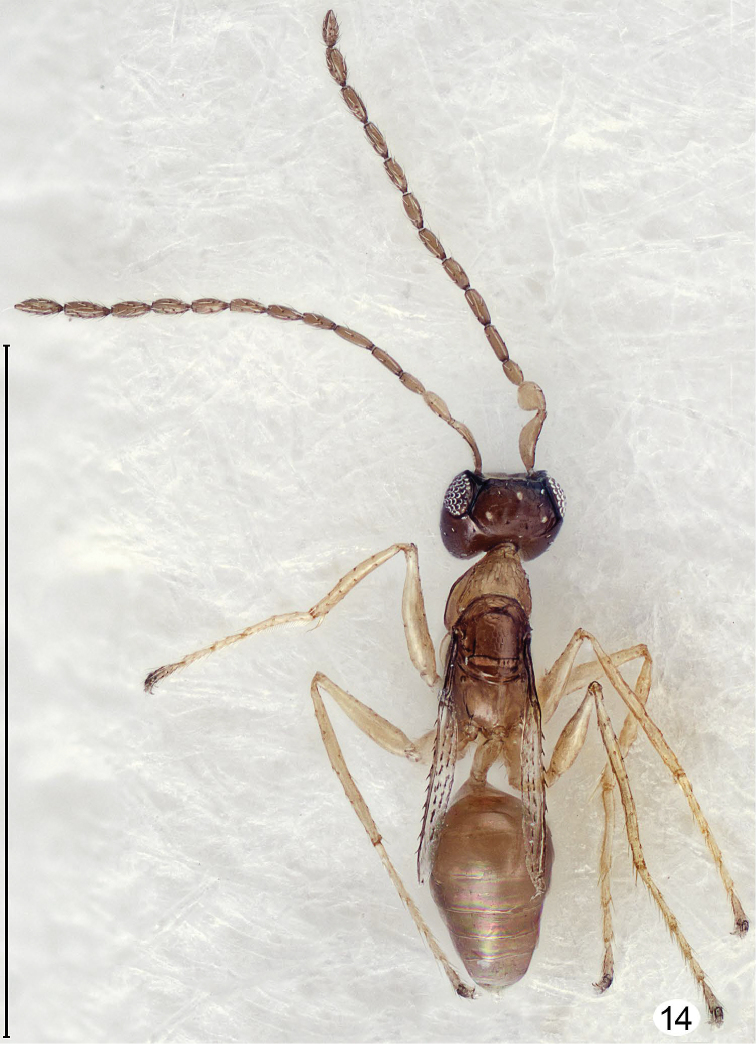
*Cremnomymar* sp., strongly brachypterous male, dorsal (forewing slightly convex, hind wing absent). Scale line = 1000 μm.

I examined forty-three specimens (not the types) of *Cremnomymar* (including the synonyms proposed above), all from Masatierra, the largest of the Juan Fernández Islands. In some of the speciesthe fore wing is fully developed and flat (Fig. 11) but in others it is more or less reduced and convex (Figs 12–14). Depending on the extent of wing reduction the mesothorax is also reduced but the placoid sensilla on the scutellum are always widely separated. The pronotal structure varies from entire, sometimes with indication of a mediolongitutinal line, to being apparently completely divided medially by a complete longitudinal carina. The propodeum in short-winged specimens changes in ways that I consider to be at most of species-level significance, from strongly carinate and with a large, sublateral tooth bearing the propodeal seta, as in fully winged *Cremnomymar* (Figs 11, 12, 32) to almost smooth (Fig. 14) but still with the propodeal seta on a bump or tooth. Therefore, I propose the above generic synonymies and transfer the included species to *Cremnomymar* as *Cremnomymar caudatum* (Ogloblin 1952), comb. n., *Cremnomymar dipteron* (Ogloblin 1957), comb. n., and *Cremnomymar kuscheli* (Ogloblin 1952), comb. n.

## Biogeography

St. Helena, with a land area of 122 km^2^, is a remote island of volcanic origin in the South Atlantic Ocean at 15°54'–16°01'S, 5°37'–5°47'W, about 1920 km W. of Africa, the nearest continent (Ashmole and Ashmole 2000). The highest elevation is 823 m and the island’s age is estimated to be 14.3 ± 1.0 million years. It has a tropical climate moderated by the Benguela Current and southeast trade winds. Two distinct physiographic zones are defined, a wet well-vegetated area above 450 m and an arid, poorly-vegetated zone below. A considerable proportion of the plant species are endemic.

The Juan Fernández islands with a total land area of 124 km^2^, almost identical in size to St. Helena, are in the South Pacific Ocean about 600 km west of Chile. Masatierra Island (Robinson Crusoe Island), at 33°38'S, 78°51'W with a land area of about 51 km^2^ and a 916 m high point is 3.8–4.2 million years old. Masafuera Island (Alejandro Selkirk Island) at 33°46'S, 80°47'W with a land area of 50 km^2^ and a 1319 m high point is 1.0–2.4 million years old. Santa Clara, the smallest island is 1 km SW of Robinson Crusoe and 5.8 million years old (Haberle 2009). Over half of the native plants species (126 of 209) are endemic. The climate is subtropical and its climate is affected by the cold Humboldt Current.

Despite being two to three times the age of Santa Clara, the oldest of the Juan Fernández Islands, the remoteness of St. Helena from the nearest continent is likely the main reason why it has only one endemic species of Mymaridae. In contrast, the Juan Fernández Islands have several endemic species, diverse enough to have been placed in separate genera by Ogloblin, who was swayed by the most notable structural differences among species of three of the four genera, i.e., wing reduction with concomitant modifications of the mesosoma. Another reason for the difference in faunas of the two groups of islands is the harsher climate on the Juan Fernández Islands (subtropical) compared to St. Helena (tropical), causing a greater selection pressure on any terrestrial organism established there and therefore a greater likelihood of diversification into several species. Two other reasons are: 1) there is greater chance of continental species reaching the Juan Fernández Island than St. Helena, so multiple introductions could account for the diversity of *Cremnomymar* species on Masatierra; and 2) the fauna (and flora) of St. Helena has been much more affected by human activity, possibly resulting in the disappearance of other species of Mymaridae that may have existed there, leaving only *Mymarilla wollastoni* among the endemic Mymaridae.

**Figure 15.**
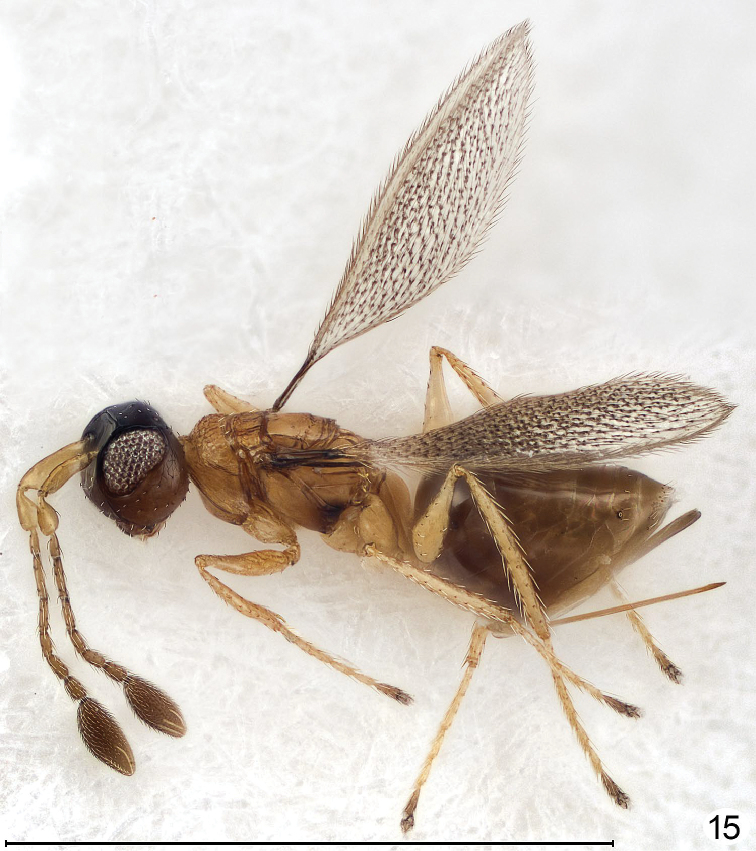
*Cremnomymar* sp., macropterous female, dorsolateral (fore wing convex, hind wing brachypterous). Scale line = 1000 μm.

**Figure 16.**
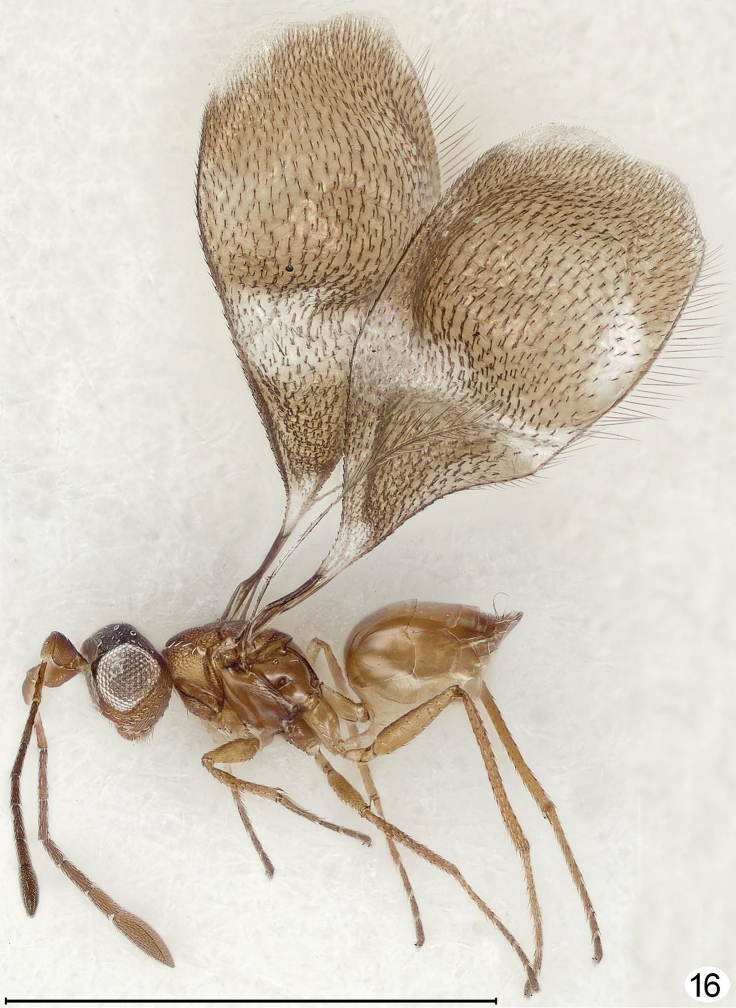
*Richteria ara*, female, lateral. Scale line = 1000 μm.

**Figures 17, 18.**
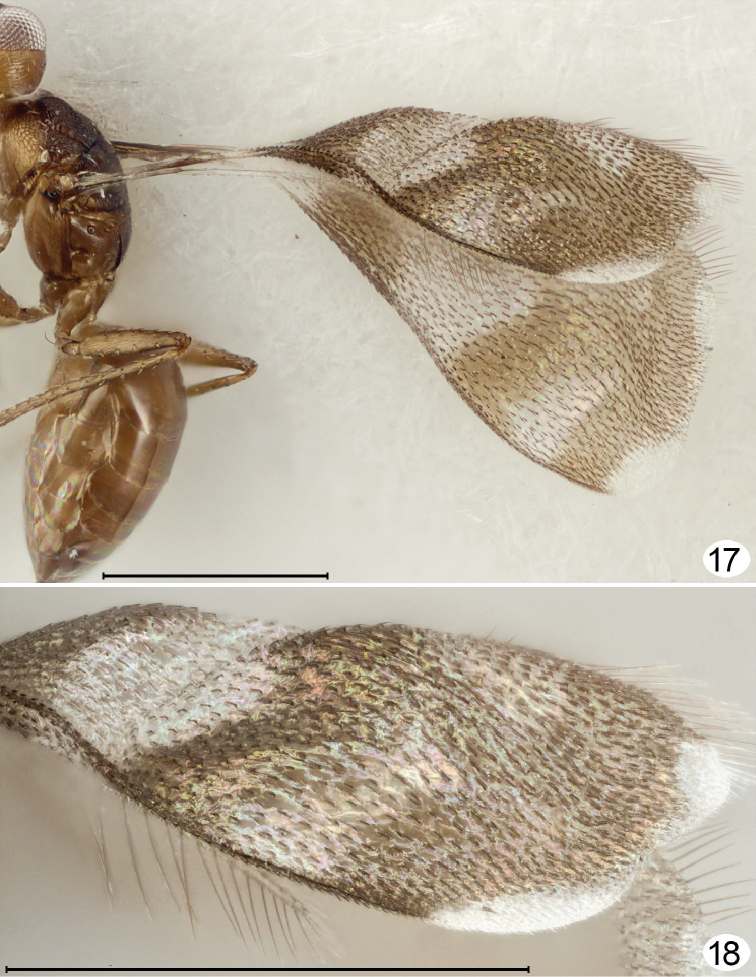
*Richteria ara*, male. **17** lateral, wings dorsoposterior **18** fore wing detail. Scale line = 500 μm.

One specimen of *Parapolynema* Fidalgo was seen, collected in Bolivia, La Paz, Sorata environs, 3200m, 20.iv.1997, L. Masner, sweeping open scrub (1 female, CNC). Fidalgo (1982, 1991) stated that *Parapolymema* is most similar to *Cremnomymar*, based on fore wing structure (Fig. 15) and propodeal seta set on a similar (as in some *Cremnomymar*), distinct protuberance. But the fore wing also is remarkably similar to that of *Richteria* Girault (Figs 16–18). The endemic Mymaridae on the Juan Fernández Islands are probably derived from specimens of *Parapolynema* that arrived there from southern South America but there seems to be a link to the New Zealand/Australian fauna and all three genera may be Gondwanan remnants.

## Wing modification in Mymaridae

The wings of Mymaridae vary considerably in shape, perhaps more than in most other families of Chalcidoidea. In a normal, fully developed (macropterous) wing the surface is flat, with the length several times greater than width and with a more or less rounded apex. Variations in shape are due partly to changes in length and width and partly to changes in outline, particularly of the posterior margin and wing apex. Depending on the genus and species a full length fore wing may become narrower so that length becomes even greater relative to width. The extremes are 2.5× as long as wide in *Paranaphoidea* Girault (Lin et al. 2007, fig. 206) and 30× as long as wide in *Cleruchus biciliatus* (Ferrière) (Ferrière 1952). Hind wing shape varies only slightly because it is generally linear to begin with. Variation in a full length hind wing is therefore mainly in width, from extremely narrow, thread-like, e.g., *Mymar* (Huber et al. 2009, fig. 55) to wide, e.g., *Paranaphoidea* (Lin et al. 2007, fig. 206); rarely the hind wing may be curved, with the anterior and posterior margins distinctly convex and concave, respectively. Despite all this variation the wings remain two dimensional. Most of the range of wing shapes and sizes is illustrated in generic treatments of Mymaridae by Subba Rao (1983) for the Oriental region, Noyes and Valentine (1989) for New Zealand, Yoshimoto (1990) for the Western Hemisphere, Huber (1997) for North America, Lin et al. (2007) for Australia, Huber et al. (2009) for the Arabian Peninsula, and Luft Albarracin et al. (2009) for Argentina.

Wing reduction (brachyptery, microptery) or complete loss (aptery) usually occurs where there a strong selection pressure against having fully developed wings. Reduction in length may be slight shorter than normal, to extremely short, at least in one sex (usually the female) of at least one species of a genus. Species with wings reduced or absent occur on all continents, mostly in species that search for host eggs in confining places where wings would be a hindrance, e.g., in soil, leaf litter, or in tubules of bracket fungi (some *Cleruchus* spp.) or in windy places, e.g., small, remote oceanic islands and high elevations on mountains. All three species (100%) of Mymaridae found on small islands (Campbell I., Auckland I., South Georgia) at more than 45° south are wingless or micropterous. Each species is placed in its own genus and each genus is evidently related to a genus on the nearest mainland or is a wingless species with winged congeners in New Zealand, and South America, respectively. For example, on South Georgia (at or near sea level) *Notomymar* is completely wingless, whereas at 4100 m in Ecuador (Yoshimoto 1990) the species there is micropterous, and at low altitude in Chile, the species (undescribed?) is/are fully winged.

On remote oceanic islands between 30°–45° S Mymaridae are reported so far only from the Juan Fernández Islands (*Cremnomymar*, 7 described spp., but some probably synonyms), Norfolk Island (*Cybomymar* Noyes & Valentine [Lin et al. 1997], 1 sp.), and Lord Howe Island (*Anagroidea* sp., new record). Wingless or short-winged species represent at least 20% of the fauna, excluding genera/species likely introduced over the past few hundred years by humans. In contrast, Mymaridae from continents south of 30° S contain a low percentage of short winged or wingless species. The exception is New Zealand (a large continental island) with 17 of 42 genera that contain at least one flightless species (Noyes and Valentine 1989: 8).

Between the tropics of Cancer and Capricorn no wingless or short winged species have yet been recorded on the relatively well studied but still poorly known (for Mymaridae) Pacific oceanic islands: Micronesia (Doutt 1955), Fiji (Huber 2009), Galapagos Islands (Huber, unpublished), Hawaiian Islands (Beardsley and Huber 2000) and, in the Atlantic Ocean, the Cape Verde Islands (Viggiani and Jesu 1995). Northern Hemisphere islands north of the tropic of Cancer are almost all in the North Atlantic Ocean. They are either too close to continents to develop endemic faunas that include short-winged or wingless species or they are too far north and therefore too cold for most insects to survive. They have generally been poorly explored, except Madeira (Graham 1979, 1983, 1988).

Curvature of the fore wing so it becomes convex or dome-shaped, i.e., it is no longer flat but clearly three-dimensional, occurs in at least one species of four genera: *Cremnomymar* Ogloblin (Figs 12, 13), *Mymarilla* (Figs 1, 7), *Parapolynema* Fidalgo (Fig. 15), so far known from Argentina and Bolivia, and *Richteria* (Figs 16–18), so far known from Australia and New Zealand. On wind-swept islands convex wings may perhaps have survival value by enveloping the posterior part of the mesosoma and the metasoma, either for greater heat absorption and retention or to reduce the chance of being blown away. But species of *Parapolynema* and *Richteria* do not necessarily occur in harsh environments so there evidently are other reasons for having a more or less convex fore wing. Interestingly, the fore wings of both genera have a double dome, in contrast to the single dome of *Mymarilla* and some *Cremnomymar*.

It would be interesting to find out if Mymaridae occur on other remote Southern Hemisphere islands, particularly those south of 30°. Collecting in a diversity of microhabitats using yellow pan traps or sifting of soil and whatever vegetation is present is required because any Mymaridae present are almost certain to be wingless or short winged.

**Figure 19.**
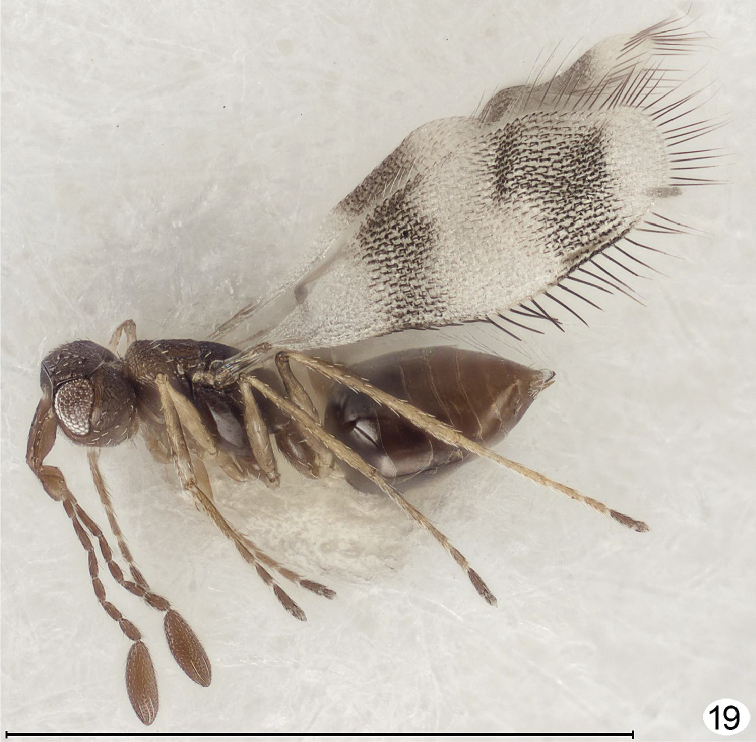
*Parapolynema* sp., female, dorsolateral. Scale line = 1000 μm.

**Figures 20–25.**
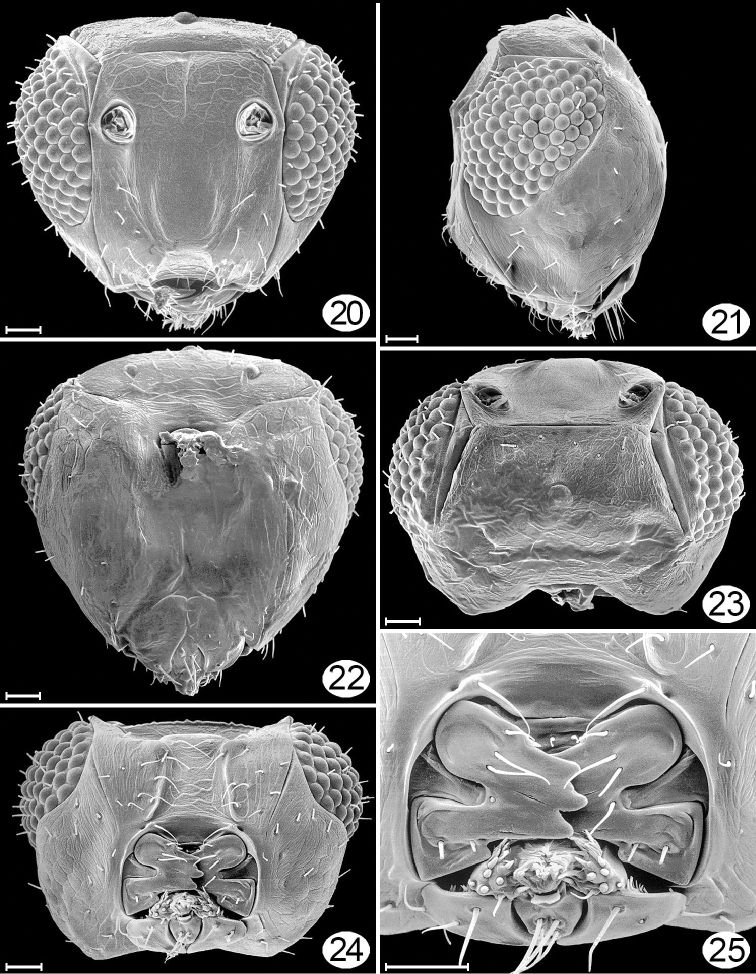
*Cremnomymar* sp. (macropterous), micrographs. **20** head, anterior **21** head, lateral **22** head, posterior (sculpture hidden by glue except laterally and dorsally) **23** head, dorsal **24** head, ventral **25** mouthparts. Scale lines = 20 μm.

**Figures 26–31.**
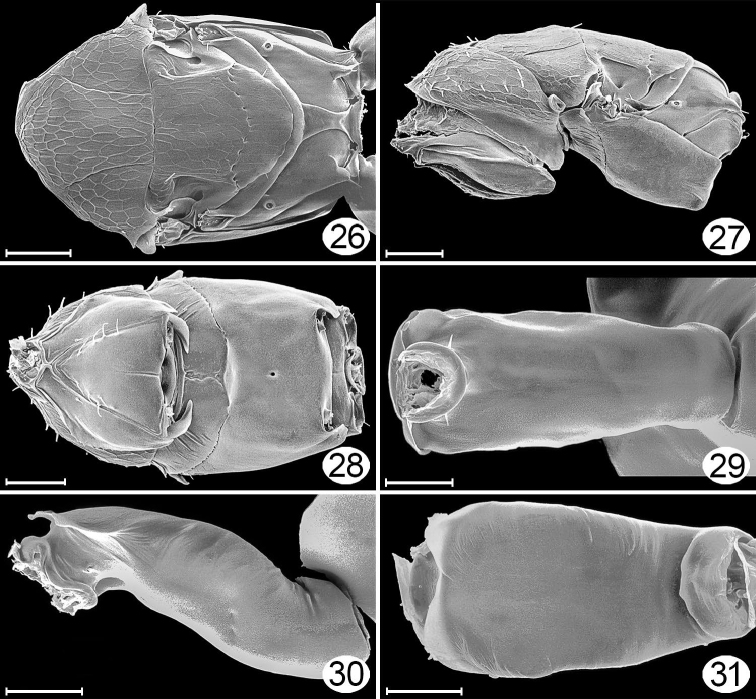
*Cremnomymar* sp. (macropterous), micrographs. **26** mesosoma (pronotum missing), dorsal **27** mesosoma, lateral **28** mesosoma, ventral **29** petiole, dorsal **30** petiole, lateral **31** petiole, ventral. Scale lines for **26–28**= 50 μm; for **29–31** = 20 μm.

**Figures 32–38.**
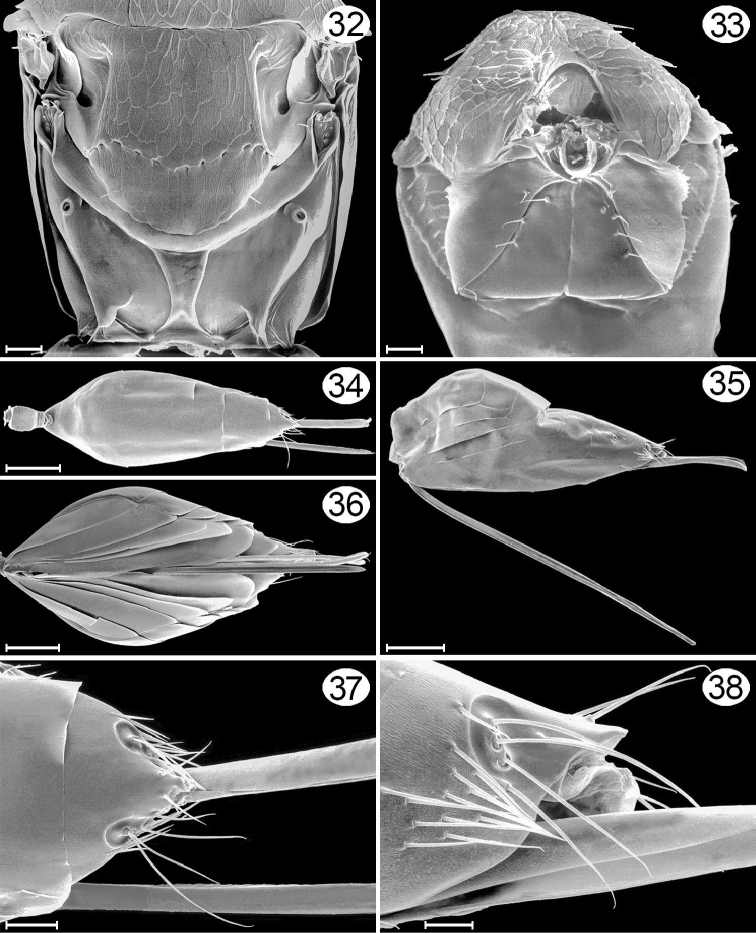
*Cremnomymar* sp. (macropterous), micrographs. **32** frenum + propodeum, dorsal **33** mesosoma, ventroanterior **34** metasoma, dorsal **35** metasoma, lateral **36** metasoma, ventral **37** apex of gaster, dorsal **38** apex of gaster, lateral. Scale lines for **34–36** = 100 μm; for **32, 33, 37, 38** = 20 μm.

## Supplementary Material

XML Treatment for Mymarilla

XML Treatment for Mymarilla wollastoni

XML Treatment for Cremnomymar
